# Postoperative recurrence of gastric cancer depends on whether the chemotherapy cycle was more than 9 cycles: Based on a retrospective and observational study of follow-up within 3 years of 843 patients

**DOI:** 10.1097/MD.0000000000028620

**Published:** 2022-02-04

**Authors:** Yifan Li, Haoliang Zhao

**Affiliations:** a Shanxi Medical University, Second Department of General Surgery, Shanxi Cancer Hospital, Taiyuan, Shanxi, People's Republic of China; b Shanxi Medical University, Department of Hepatobiliary Surgery, Shanxi Bethune Hospital, Taiyuan, Shanxi, People's Republic of China.

**Keywords:** chemotherapy cycle, gastric cancer, recurrence, risk factors

## Abstract

We retrospectively reviewed the medical records of patients with pathologically confirmed gastric cancer/adenocarcinoma who underwent curative surgical resection follow-up within 3 years at Shanxi cancer hospital between 2002 and 2020. The clinicopathologic parameters explored included gender, age at surgery, vascular invasion, neural invasion, Tumor infiltration depth (T stage), N stage, TNM stage, chemotherapy, Lauren classification, maximum diameter of tumor, type of gastrectomy, tumor location and survival data.

With a median follow-up of 29 months (range 0–36 months), the ratio of patients with recurrence was 26.80% (n = 226) and the death rate of patients was 45.31% (n = 382) in this period. According to the results of univariate analysis, gender (*P* = .014), age at surgery (*P* = .010), vascular invasion (*P* = .000), neural invasion (*P* = .000), T stage (*P* = .000), N stage (*P* = .000), TNM stage (*P* = .000), chemotherapy cycle (*P* = .000), lauren classification (*P* = .000), maximum diameter of tumor (*P* = .000), type of gastrectomy (*P* = .000) were independent risk factors of recurrence of follow-up within 3 years. From the multivariate analysis by logistic regression showed that TNM Stage (*P* = .002), chemotherapy cycle (*P* = .000) were risk factors of recurrence of follow-up within 3 years. Univariate analysis of survival by Kaplan–Meier showed that gender (*P* = .038), vascular invasion (*P* = .000), neural invasion (*P* = .000), maximum diameter of tumor (*P* = .000), Lauren classification (*P* = .000), T stage (*P* = .000), N stage (*P* = .000), TNM Stage (*P* = .000) and type of gastrectomy (*P* = .000) were key factors linked to overall survival of follow-up within 3 years. The results of the multivariate analysis by Cox regression were clearly presented that T Stage (*P* = .000), TNM stage (*P* = .001), maximum diameter of tumor (*P* = .001) were key factors of overall survival of follow-up within 3 years.

TNM Stage, chemotherapy cycle were closely related to recurrence and of follow-up within 3 years. More than 9 cycles of chemotherapy was able to reduce the probability of recurrence. T Stage, TNM stage, maximum diameter of tumor were independent factors associated with overall survival of gastric cancer of follow-up within 3 years. For maximum diameter of tumor, the probability of death of more than 6 cm was 1.317 times less than 6 cm within 3 years of follow-up.

## 1 Introduction

Gastric cancer is the fifth most common malignant tumor in the world and the third most common cause of malignant tumor-related death.^[1]^ Though the overall survival (OS) of gastric cancer patients has improved with the development of standardized D2 lymphadenectomy^[2]^ and subsequent adjuvant chemotherapy in recent years,^[3,4]^ the long-term survival rate is still unsatisfactory. Recurrencies the main cause of disease-related death.^[5]^ In our retrospective study, we found that the ratio of recurrence was 26.80% (n = 43) and the mortality rate was 46.07% (n = 94) within 1 year of follow-up. More than 1 year and less than 3 years, the ratio of recurrence and was 28.64% (n = 183) and the mortality rate was 45.07% (n = 288). In present study, we aimed to clarify the prognostic factors associated with recurrence after curative resection within 3 years of follow-up, which would help clinicians perform the appropriate treatment in order to improve OS.

## 2 Methods section

### 2.1 Patients

Between May 2002 and December 2020, a total of 1715 patients with gastric cancer/adenocarcinoma after radical resection were selected from Shanxi cancer hospital, Shanxi, China. All of these patients underwent lymphadenectomy higher than D2 (complete removal of group 1 and 2 lymph nodes). All patients’ clinicopathological characteristics, including gender, age at surgery, vascular invasion, neural invasion, Tumor infiltration depth (T stage), N stage, TNM stage (according to the 8th Edition of the American Joint Committee on Cancer staging manual),^[6]^ chemotherapy, Lauren classification, maximum diameter of tumor, type of gastrectomy, tumor location and survival data, were retrospectively reviewed based on operative notes, medical records and telephone follow-ups. In our group, we selected 843 patients with follow-up within 3 years, all of these patients follow-up less than 3 years, including patients who died within 3 years and patients who alive and follow-up ended within 3 years.

### 2.2 Postoperative follow-up

The patients were followed closely until December 2020, the average length of follow-up was 38.32 (range 1–156) months. Follow-up assessments were performed every 3 months for the first 1 years after surgery, every 6 months for 2 to 5 years, and yearly thereafter. Routine follow-up consisted of physical examination, laboratory tests, chest radiography, abdominopelvic ultrasonography, computed tomography and magnetic resonance imaging. OS was defined as the time from curative resection of gastric cancer to death or the last follow-up time. All of methods and follow-up information reviewed and approved by the Medical Ethics committee of Shanxi cancer hospital.

### 2.3 Statistical analysis

All data were analyzed with SPSS 23.0 (SPSS Inc., Chicago, IL) software. Categorical variables were analyzed with a Chi-Squared test and Fisher exact test. Univariate analyses were performed with the Kaplan–Meier method. Continuous data, expressed as the mean ± standard deviation (SD), were compared by Student *t* test. In the multivariate analysis, logistic regression analysis was used to evaluate the risk factors of recurrence and cox regression analysis was applied to identify independent risk factors associated with survival. Multiple linear regression was applied to screen out the risk factors of recurrence and stepwise regression was used to select the most factor linked to recurrence. Survival analyses and curves were established with the Kaplan–Meier method and compared with the Log rank test. *P* < .05 was considered statistically significant.

## 3 Results

### 3.1 Clinical characteristics

Of all these patients (n = 1715), the ratio of patients with recurrence was 23.90% (n = 410) and the death rate of patients was 38.08% (n = 653). The mean age was 58.74 ± 10.04 years old. In this group, 79.53% were female (n = 1364) and 20.47% were is female (n = 351). The average of follow-up was 38.32 ± 22.50 months. The 1 -, 3 -, 5 - year survival rates of these patients were 94.14%, 69.53%, 37.71% respectively. With regard to tumor location, including upper (n = 866), middle (n = 285), lower (n = 384) of the stomach and multiple (n = 16) site. About type of gastrectomy, including proximal gastrectomy (n = 166), distal gastrectomy (n = 555), total gastrectomy (n = 992) and PPG (n = 2). The average of maximum diameter of tumor was 5.03 ± 2.60 cm. The mean of chemotherapy cycle was 9.83 ± 3.80 cycles. In these patients (n = 1715), we selected patients with follow-up within 3 years (n = 843), the ratio of patients with recurrence was 26.80% (n = 226) and the death rate of patients was 45.31% (n = 382) in this period.

### 3.2 Risk factors for recurrence of follow-up within 3 years

The univariate analysis of postoperative recurrence was presented in Table 1 and these data was analyzed by Chi-Squared test and Fisher exact test. According to the results of univariate analysis, gender (*P* = .014), age at surgery (*P* = .010), vascular invasion (*P* = .000), neural invasion (*P* = .000), T stage (*P* = .000), N stage (*P* = .000), TNM stage (*P* = .000), chemotherapy cycle (*P* = .000), Lauren classification (*P* = .000), maximum diameter of tumor (*P* = .000), type of gastrectomy (*P* = .000) were independent risk factors of recurrence of follow-up within 3 years. In addition, tumor location was not significantly different between recurrence group and no-recurrence group (*P* > .05).

**Table 1:** The univariate analysis of postoperative recurrence and metastasis.

	Recurrence and metastasis		*P*
Risky factors	Yes	NO	Univariate
Gender			
Male	166	501	χ^2^ = 6.011
Female	60	116	*P* = .014
Age (yr)			
≤58	108	234	χ^2^ = 6.637
>58	118	383	*P* = .010
T-stage			
T1	9	108	χ^2^ = 54.875
T2	4	22	*P* = .000
T3	69	257	
T4	144	230	
N stage			
N0	18	211	χ^2^ = 85.761
N1	27	112	*P* = .000
N2	31	83	
N3	150	211	
TNM stage			
I	7	120	χ^2^ = 89.081
II	26	168	*P* = .000
III	167	313	
IV	26	16	
Tumor location^∗^			
Upper	116	338	χ^2^ = 3.076
Middle	47	110	*P* = .365
Lower	63	163	
Multiple	0	6	
Type of gastrectomy			
Proximal	9	50	χ^2^ = 11.447
Distal	59	168	*P* = .000
Total	188	369	
Vascular invasion			
Negative	47	288	χ^2^ = 46.267
Positive	179	329	*P* = .000
Neural invasion			
Negative	72	316	χ^2^ = 24.949
Positive	154	301	*P* = .000
Lauren classification			
Intestinal	38	232	χ^2^ = 46.622
Diffuse	129	205	*P* = .000
Mixed	59	180	
Maximum diameter of tumor (cm)			
<6	105	388	χ^2^ = 18.378
≥6	121	229	*P* = .000
Chemotherapy Cycle			
<9 cycle	198	15	χ^2^ = 221.678
≥9 cycle	214	416	*P* = .000

∗ Fisher exact test.

For multiple experimental groups, T stage, N stage, TNM stage, Type of gastrectomy, Lauren classification were closely associated with recurrence, so we performed pairwise comparisons for these risk factors (Table 2). The results of pairwise comparisons of T stage is that the difference of T1vsT3 (*P* = .001), T1vsT4 (*P* = .000), T2vsT4 (*P* = .020) T3vsT4 (*P* = .000) was significantly between each other. For N stage, the otherness between each stage was apparently (*P* < .05) except for N1vsN2 (*P* = .176). With regard to TNM Stage and Lauren classification, there were significant differences between their each stage. About the type of gastrectomy, the ratio of recurrence of proximal gastrectomy versus total gastrectomy (*P* = .000), distal gastrectomy vs total gastrectomy (*P* = .034) were different between each other. In other words, the ratio of recurrence of total gastrectomy is higher than proximal gastrectomy and distal gastrectomy.

**Table 2:** Pairwise comparisons of multiple experimental groups.

Risky factors	χ^2^	*P*
T-stage		
T1 vs T2	χ^2^ = 1.523	.255
T1 vs T3	χ^2^ = 10.774	.001
T1 vs T4	χ^2^ = 39.439	.000
T2 vs T3	χ^2^ = 0.490	.619
T2 vs T4	χ^2^ = 5.574	.020
T3vsT4	χ^2^ = 24.730	.000
N stage		
N0 vs N1	χ^2^ = 10.777	.001
N0 vs N2	χ^2^ = 23.231	.000
N0 vs N3	χ^2^ = 78.091	.000
N1 vs N2	χ^2^ = 2.139	.176
N1 vs N3	χ^2^ = 21.486	.000
N2 vs N3	χ^2^ = 7.573	.006
TNM stage		
I vs II	χ^2^ = 5.181	.023
I vs III	χ^2^ = 42.105	.000
I vs IV	χ^2^ = 63.876	.000
II vs III	χ^2^ = 30.932	.000
II vs IV	χ^2^ = 47.280	.000
III vs IV	χ^2^ = 12.183	.001
Lauren classification		
Intestinal vs diffuse	χ^2^ = 44.979	.000
Intestinal vs mixed	χ^2^ = 9.256	.002
Diffuse vs mixed	χ^2^ = 12.274	.001
Type of gastrectomy		
Proximal vs distal	χ_2_ = 2.979	.089
Proximal vs total	χ_2_ = 8.392	.005
Distal vs total	χ_2_ = 2.979	.034

From the multivariate analysis analyzed by logistic regression showed that TNM Stage (*P* = .002), chemotherapy cycle (*P* = .000) were risk factors of recurrence of follow-up within 3 years (Table 3). As for N stage, N Stage and recurrence of follow-up within 3 years were positive correlation. Furthermore, the difference of N0 VS N3 (*P* = .029) was significant, that means the ratio of recurrence of N3 was higher than N0. Moreover, TNM Stage and recurrence of follow-up within 3 years were positive correlation, chemotherapy cycle and recurrence of follow-up within 3 years were negative correlation. Namely, regarding TNM Stage, as the stage increases, the likelihood of recurrence increases. Inadequate chemotherapy cycle may be a risk factor for recurrence of follow-up within 3 years. The value of odds ratio for chemotherapy cycle means that the probability of recurrence more than 9 cycles is 0.296 times as much as less than 9 cycles.

**Table 3:** Multivariate analysis of postoperative recurrence and metastasis analysed by logistic regression.

Risky factors	*B*	SE	Wald	df	*P*	Exp(B)	95% CI
Gender							
Male vs Female	−0.199	0.216	0.854	1	.355	0.819	0.537–1.250
Age (yr)	−0.011	0.009	1.504	1	.220	0.989	0.972–1.006
T-stage	3.873	3	0.275				
T1 vs T2	−0.660	0.885	0.556	1	.456	0.517	0.091–2.929
T1 vs T3	−1.109	0.874	1.609	1	.205	0.330	0.060–1.830
T1 vs T4	−0.768	0.890	0.744	1	.388	0.464	0.081–2.655
N stage			5.619	3	.132		
N0 vs N1	0.530	0.409	1.681	1	.195	1.699	0.763–3.783
NO vs N2	0.832	0.536	2.404	1	.121	2.297	0.803–6.571
N0 vs N3	1.119	0.512	4.773	1	.029	3.062	1.122–8.354
TNM Stage			14.547	3	.002		
I vs II	1.050	0.913	1.323	1	.250	2.859	0.477–17.128
I vs III	1.381	1.074	1.652	1	.199	3.978	0.484–32.672
I vs IV	2.500	1.071	5.446	1	.020	12.187	1.492–99.517
Vascular invasion							
Negative vs positive	0.252	0.229	1.219	1	.270	1.287	0.822–2.014
Neural invasion							
Negative vs positive	−0.044	0.201	0.048	1	.827	0.957	0.645–1.419
Lauren classification			1.076	2	.584		
Intestinal vs diffuse	0.237	0.249	0.900	1	.343	1.267	0.777–2.066
Mixed vs intestinal	0.073	0.260	0.079	1	.779	1.076	0.646–1.792
Maximum diameter of tumor (cm)							
<6 vs ≥6	0.176	0.191	0.851	1	.356	1.193	0.820–1.735
Chemotherapy cycle							
<9 cycle vs ≥9 cycle	−1.216	0.190	41.119	1	.000	0.296	0.204–0.430
Type of gastrectomy			0.729	2	.694		
Proximal vs distal	0.135	0.446	0.092	1	.761	1.145	0.478–2.744
Proximal vs total	−0.049	0.434	0.013	1	.910	0.952	0.407–2.227

B = regression coefficient, CI = confidence interval, *df* = degree of freedom, Exp(B) = odds ratio, SE = standard error.

On the basis of Table 4, that clearly displayed that N stage (*P* = .004), TNM Stage (*P* = .002), chemotherapy cycle (*P* = .000) were risk factors of recurrence of follow-up within 3 years. Furthermore, N stage, TNM Stage and recurrence of follow-up within 3 years were positive correlation, chemotherapy cycle and recurrence of follow-up within 3 years were negative correlation. According to absolute value of beta, chemotherapy cycle has the greatest influence on recurrence of follow-up within 3 years due to the maximum absolute value of 3 of these risk factors of recurrence. The value of these 3 factors of VIF no more than 3, that means that there is no collinearity problem between these independent variables.

**Table 4:** Multivariate analysis of postoperative recurrence and metastasis used by stepwise regression of multiple linear regression.

Risky factors	*B*	SE	Beta	*t*	Sig	Tolerance	VIF
N stage	0.053	0.018	0.152	2.918	0.004	0.368	2.717
Chemotherapy Cycle	−0.238	0.033	−0.234	−7.321	0.000	0.979	1.022
TNM Stage	0.088	0.028	0.161	3.110	0.002	0.371	2.695

B = regression coefficient, *Beta* = standardized coefficients, SE = standard error, sig = significance, VIF = variance inflation factor.

### 3.3 Survival analysis

**Figure 1:**
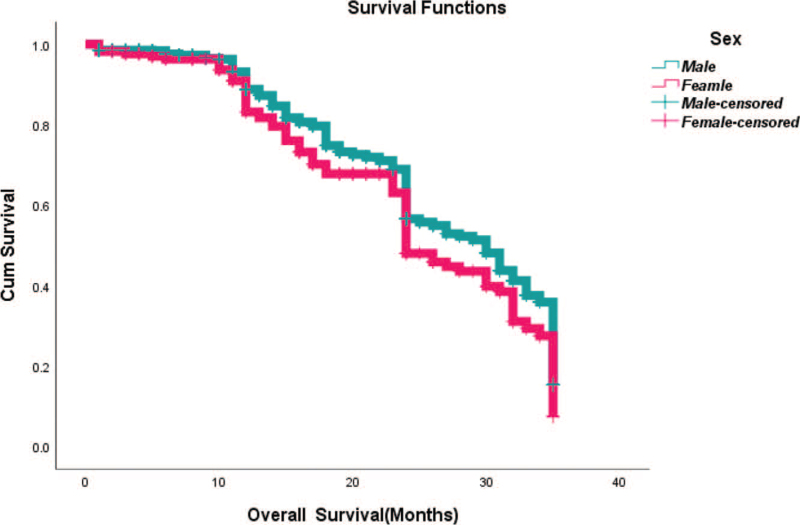
OS curves for sex.

**Figure 2:**
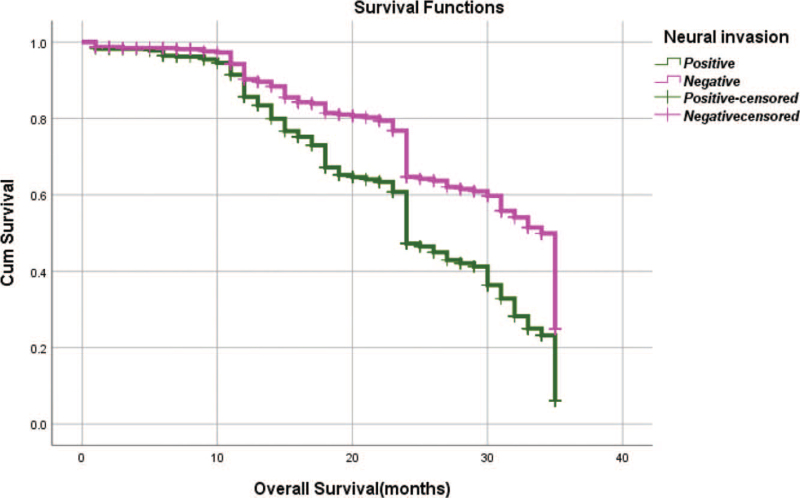
OS curves for vascular invasion.

**Figure 3:**
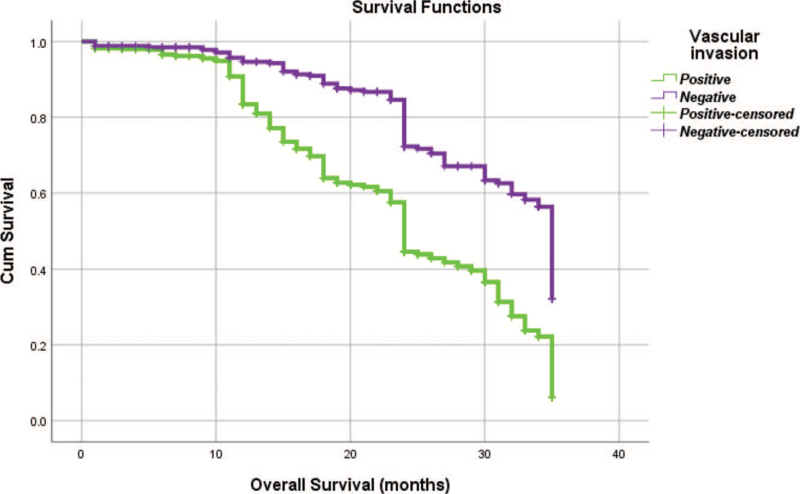
OS curves for neural invasion.

**Figure 4:**
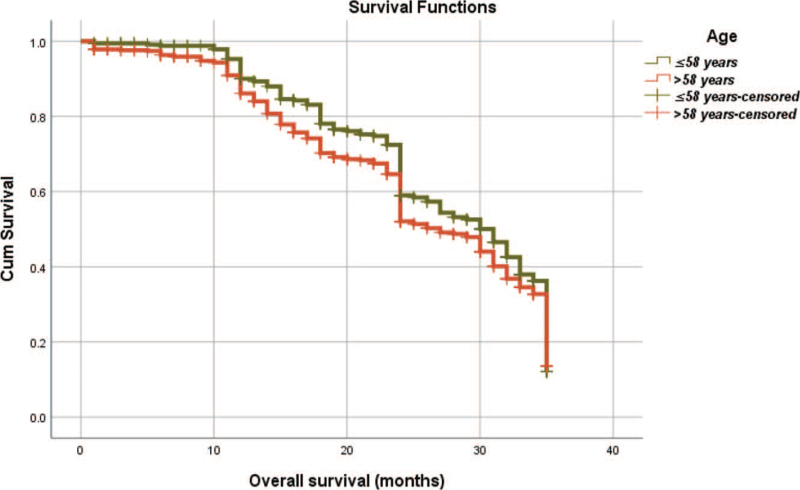
OS curves for age.

**Figure 5:**
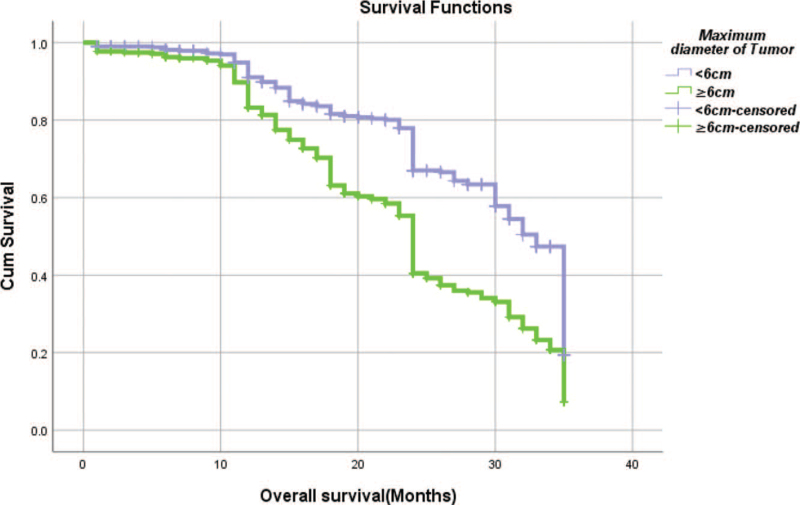
OS curves for maximum diameter of tumor.

**Figure 6:**
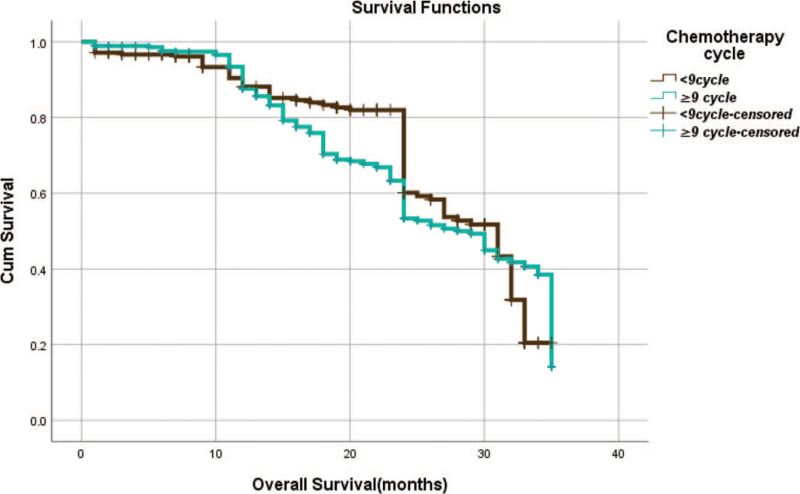
OS curves for chemotherapy cycle.

**Figure 7:**
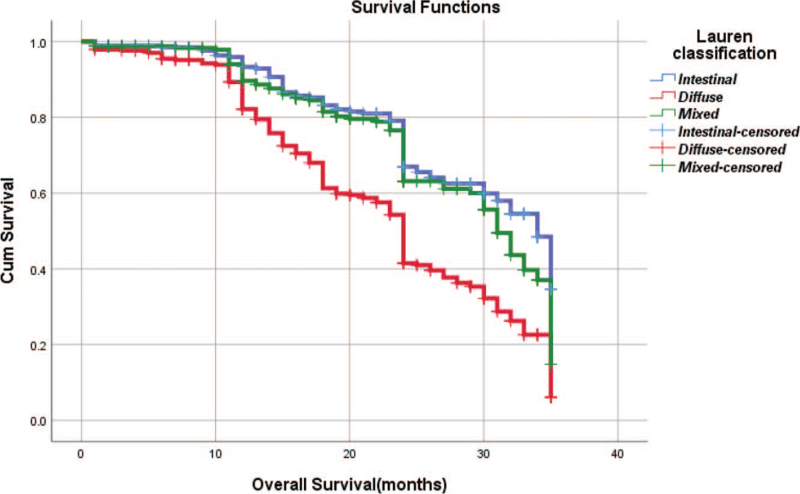
OS curves for Lauren classification.

**Figure 8:**
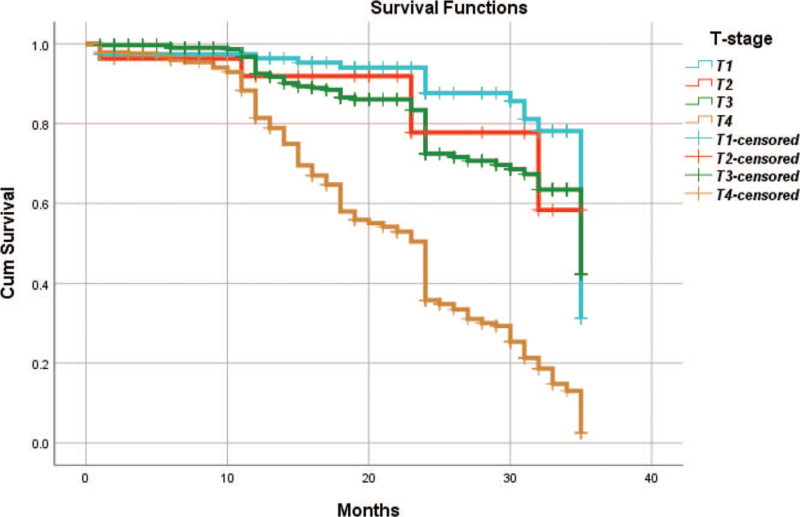
OS curves for T stage.

**Figure 9:**
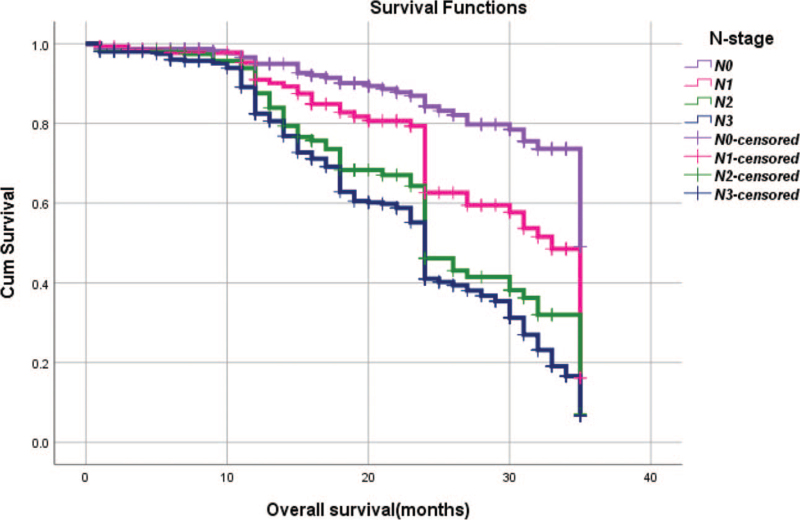
OS curves for N stage.

**Figure 10:**
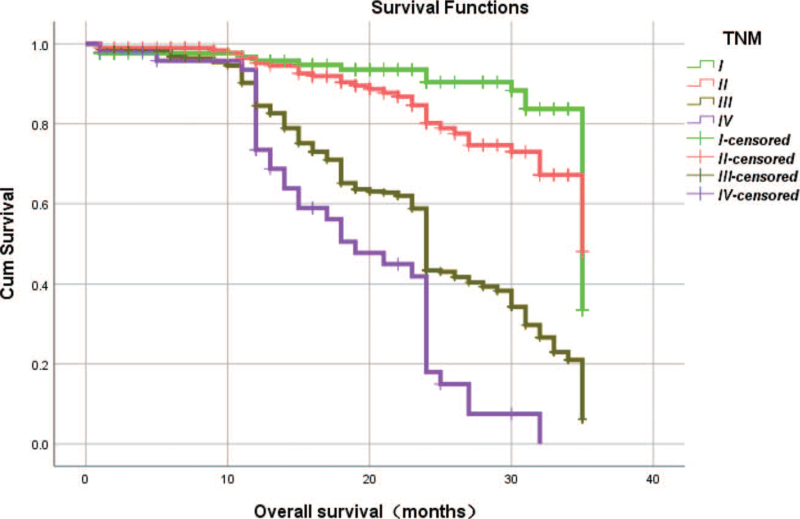
OS curves for TNM.

**Figure 11:**
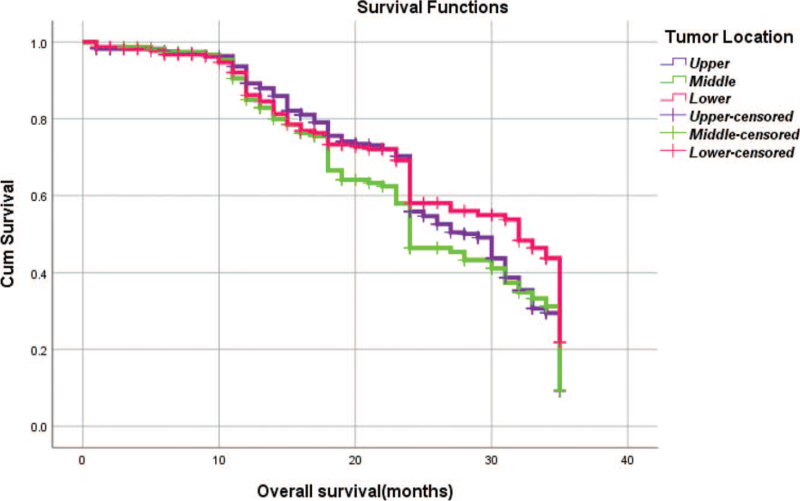
OS curves for tumor location.

**Figure 12:**
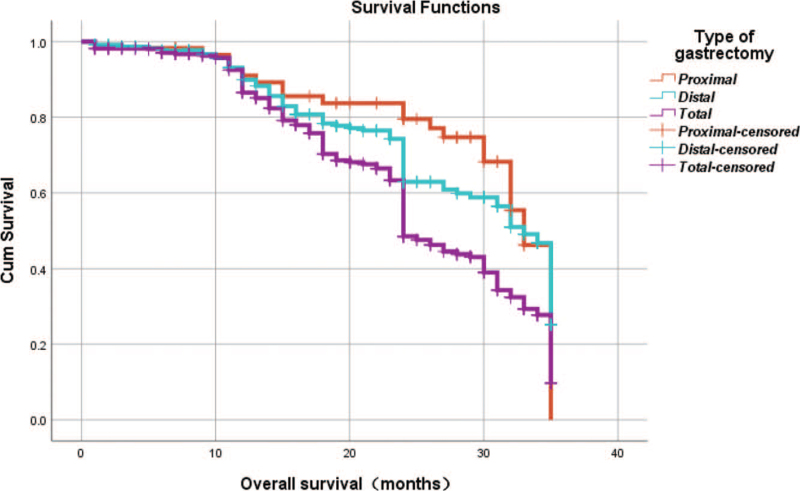
OS curves for type of gastrectomy Word count: 4310.

From Table 5, we can clearly see that gender (*P* = .038), vascular invasion (*P* = .000), neural invasion (*P* = .000), maximum diameter of tumor (*P* = .000), Lauren classification (*P* = .000), T stage (*P* = .000), N stage (*P* = .000), TNM Stage (*P* = .000) and type of gastrectomy (*P* = .000) were risk factors linked to overall survival of follow-up within 3 years. The overall median survival time of this group was 29 months. The median survival time of male was 30 months and female was 24 months (Fig. 1). Compared with the positive of vascular invasion and neural invasion, median survival time of negative of vascular invasion and neural invasion was 35 months and 34 months, respectively (Figs. 2 and 3). The median survival time of patients under 58 years old was 31 months and patients over 58 years old was 27 months (Fig. 4). About maximum diameter of tumor of median survival time, less than 6 cm was 33 months and more than 6 cm was 24 months (Fig. 5). The median survival time of chemotherapy cycle less than 9 cycles and more than 9 cycles was similar, was 31 and 28 months, (Fig. 6). As far as Lauren classification was concerned, intestinal type, diffuse type and mixed type of median survival time was 34, 24, 31 months singly (Fig. 7). As for T stage, T1, T2, T3, and T4 of median survival time was 35, 35, 35, 24 months respectively (Fig. 8). About of N stage, the median survival time of N0, N1, N2, N3 was 35, 33, 24, 24 months severally (Fig. 9). Concern to TNM Stage, I, II, III, IV of median survival time was 35, 35, 24, 19 months respectively (Fig. 10). With regard to tumor location, 3 of these of median survival time was comparable, upper was 29 month, middle was 24 months, lower was 32 months (Fig. 11). As to type of gastrectomy, proximal gastrectomy, distal gastrectomy and total gastrectomy of median survival time was 33, 33, 24 months respectively (Fig. 12).

**Table 5:** The univariate analysis of overall survival by Kaplan–Meier.

	Mean			Median			
Risky factors	survival time	SE	95% CI	Survival time	SE	95% CI	*P*
Gender							.038
Male	26.365	0.396	25.588–27.142	30.000	1.215	27.618–32.382	
Female	24.644	0.829	23.019–26.270	24.000	0.999	22.043–25.957	
Vascular invasion							.000
Positive	23.805	0.459	22.905–24.705	24.000	0.195	23.617–24.383	
Negative	29.753	0.503	28.768–30.739	35.000	0.325	34.364–35.636	
Neural invasion							.000
Positive	24.307	0.478	23.370–25.244	24.000	0.516	22.988–25.012	
Negative	28.263	0.517	27.251–29.275	34.000	0.513	32.994–35.006	
Age							.055
≤58 yr	27.055	0.528	26.021–28.089	31.000	1.438	28.181–33.819	
>58 yr	25.311	0.482	24.367–26.255	27.000	0.915	25.206–28.794	
Maximum diameter of Tumor							.000
<6cm	28.304	0.452	27.418–29.191	33.000	0.459	32.100–33.900	
≥6cm	23.278	0.539	22.223–24.334	24.000	0.200	3.608–24.392	
Chemotherapy Cycle							.742
<9 cycle	26.631	0.683	25.293–27.970	31.000	1.566	27.930–34.070	
≥9 cycle	25.848	0.418	25.030–26.667	28.000	0.913	26.211–29.789	
Lauren classification							.000
Intestinal	28.613	0.599	27.438–29.788	34.000	0.942	32.153–35.847	
Diffuse	23.143	0.560	22.045–24.241	24.000	0.375	23.265–24.735	
Mixed	27.737	0.650	26.462–29.012	31.000	0.781	29.469–32.531	
T stage							.000
T1	32.382	0.721	30.967–33.796	35.000	1.105	32.834–37.166	
T2	30.410	1.845	26.793–34.027	35.000^∗^			
T3	29.990	0.550	28.913–31.068	35.000	1.288	32.475–37.525	
T4	22.005	0.485	21.054–22.956	24.000	0.545	22.931–25.069	
N stage							.000
N0	31.396	0.588	30.244–32.548	35.000^∗^			
N1	28.177	0.866	26.479–29.875	33.000	0.768	31.494–34.506	
N2	24.801	0.944	22.951–26.652	24.000	0.776	22.480–25.520	
N3	23.131	0.523	22.106–24.157	24.000	0.203	23.602–24.398	
TNM stage							.000
I	32.713	0.682	1.375–34.050	35.000	1.468	32.122–37.878	
II	30.807	0.665	29.503–32.110	35.000^∗^			
III	23.827	0.456	22.932–24.721	24.000	0.166	23.675–24.325	
IV	19.024	1.122	16.825–21.224	19.000	2.813	13.487–24.513	
Tumor location							.090
Upper	26.039	0.472	25.113–26.964	29.000	1.447	26.164–31.836	
Middle	24.730	0.825	23.113–26.347	24.000	1.540	20.981–27.019	
Lower	26.693	0.740	25.243–28.143	32.000	2.619	26.866–37.134	
Type of gastrectomy							.000
Proximal	29.450	1.201	27.096–31.804	33.000	0.606	31.813–34.187	
Distal	27.709	0.700	26.337–29.081	33.000	0.622	31.781–34.219	
Total	24.933	0.437	24.076–25.789	24.000	0.542	22.937–25.063	
Overall	26.026	0.358	25.324–26.727	29.000	0.677	27.673–30.327	

∗ T2, N0, II of median can not estimate because all of patients with T2, N0, II were alive in 3 years.

**Table 6:** Pairwise comparisons of multiple experimental groups by Kaplan–Meier.

Risky factors	χ^2^	*P*
T-stage		
T1 vs T2	χ^2^ = 0.812	.368
T1 vs T3	χ^2^ = 4.605	.302
T1 vs T4	χ^2^ = 79.587	.000
T2 vs T3	χ^2^ = 0.064	.800
T2 vs T4	χ^2^ = 12.558	.000
T3 vs T4	χ^2^ = 110.363	.000
N stage		
N0 vs N1	χ^2^ = 12.570	.000
N0 vs N2	χ^2^ = 42.269	.000
N0 vs N3	χ^2^ = 87.367	.000
N1 vs N2	χ^2^ = 7.226	.007
N1 vs N3	χ^2^ = 23.547	.000
N2 vs N3	χ^2^ = 2.944	.086
TNM stage		
I vs II	χ^2^ = 3.162	.075
I vs III	χ^2^ = 62.739	.000
I vs IV	χ^2^ = 90.699	.000
II vs III	χ^2^ = 58.664	.000
II vs IV	χ^2^ = 76.460	.000
III vs IV	χ^2^ = 13.893	.000
Lauren classification		
Intestinal vs diffuse	χ^2^ = 43.862	.000
Intestinal vs mixed	χ^2^ = 2.319	.128
Diffuse vs mixed	χ^2^ = 23.594	.000
Tumor location		
Upper vs middle	χ^2^ = 1.094	.296
Upper vs lower	χ^2^ = 2.302	.129
Middle vs lower	χ^2^ = 4.735	.030
Type of gastrectomy		
Proximal vs distal	χ^2^ = .0.609	.435
Proximal vs total	χ^2^ = 10.315	.000
Distal vs total	χ^2^ = 13.687	.030

The results of pairwise comparisons of T stage is that the difference of T1vsT4 (*P* = .000), T2 versus T4 (*P* = .000) T3 versus T4 (*P* = .000) was significantly between each other. For N stage, the difference between each stage were apparently (*P* < .05) except for N2vsN3 (*P* = .086). With regard to TNM Stage, there were significant differences between their each stage besides I vs II (*P* = .075). As for Lauren classification, intestinal versus diffuse (*P* = .000), diffuse versus mixed (*P* = .000) were different between each other. That means that the survival rate of diffuse type were higher than intestinal type and mixed type. About tumor location, only middle versus lower (*P* = .030) was significantly between each other. About the type of gastrectomy, the survival rate of proximal gastrectomy vs total gastrectomy (*P* = .000), distal gastrectomy vs total gastrectomy (*P* = 0.030) were different between each type of gastrectomy. In other words, the survival rate of total gastrectomy is higher than proximal gastrectomy and distal gastrectomy. The results of the multivariate analysis analyzed by Cox regression were clearly showed in Table 7. The outcome were that T Stage (*P* = .000), TNM stage (*P* = .001), maximum diameter of tumor (*P* = .001) were risk factors of OS of follow-up within 3 years. Moreover, TNM Stage, maximum diameter of tumor and OS of follow-up within 3 years were positive correlation. Maximum diameter of tumor may be a risk factor for OS of follow-up within 3 years. According to the hazard ratio of maximum diameter of tumor, the probability of death of more than 6 cm was 1.317 times as ≥6 cm.

**Table 7:** Multivariate analysis of overall survival analysed by COX regression.

Risky factors	*B*	SE	Wald	df	*P*	Hazard ratio	95% CI
Gender							
Male vs Female	−0.111	0.128	0.748	1	.387	0.895	0.696–1.151
Age (yr)	0.013	0.005	6.020	1	.014	1.013	1.003–1.024
T-stage			30.657	3	.000		
T1 vs T2	−0.001	0.570	0.000	1	.999	0.999	0.327–3.056
T1 vs T3	−0.620	0.517	1.434	1	.231	0.538	0.195–1.483
T1 vs T4	0.229	0.518	0.195	1	.658	1.258	0.455–3.474
N stage			0.258	3	.968		
N0 vs N1	−0.005	0.296	0.000	1	.988	0.995	0.557–1.779
NO vs N2	0.009	0.338	0.001	1	.980	1.009	0.520–1.955
N0 vs N3	0.067	0.323	0.043	1	.836	1.069	0.567–2.015
TNM stage			15.473	3	.001		
I vs II	0.821	0.560	2.146	1	.143	2.272	0.758–6.814
I vs III	1.263	0.653	3.744	1	.053	3.537	0.984–12.717
I vs IV	1.802	0.642	7.882	1	.005	6.060	1.723–21.318
Vascular invasion							
Negative vs Positive	0.257	0.143	3.217	1	.073	1.294	0.976–1.714
Neural invasion							
Negative vs Positive	0.014	0.124	0.013	1	.910	1.014	0.795–1.293
Lauren classification			2.484	2	.289		
Intestinal vs Diffuse	0.163	0.147	1.224	1	.268	1.177	0.882–1.571
Mixed vs Intestinal	−0.029	0.163	0.033	1	.856	0.971	0.706–1.336
Maximum diameter of Tumor (cm)							
<6 vs ≥6	0.276	0.112	6.086	1	.014	1.317	1.058–1.640
Chemotherapy Cycle							
<9 cycle vs ≥9 cycle	0.150	0.119	1.582	1	.208	1.162	0.920–1.468
Type of gastrectomy			0.246	2	.884		
Proximal vs Distal	0.120	0.248	0.235	1	.628	1.128	0.693–1.835
Proximal vs Total	0.109	0.235	0.214	1	.644	1.115	0.703–1.767

B = regression coefficient, CI = confidence interval, df = degree of freedom, SE = standard error.

## 4 Discussion

Most studies had noted that curative resection for gastric cancer focused largely on early gastric cancer or advanced gastric cancer. With regard to early gastric cancer, there were many articles on the risk factors of recurrence of early gastric cancer in Japan, Korea, and China. Hee Jun Choi^[7]^ reported that among the 8753 patients of T1N0M0 gastric cancer 95 patients (1.1%) experienced tumor recurrence; this included 31 remnant, 27 hematogenous, 9 lymph nodal, 5 peritoneal, and 23 multiple-site recurrences. In multivariate analysis, older age, male gender, tumor depth and venous invasion were independent risk factors for tumor recurrence in Korea. In another cohort study in Korea,^[8]^ age over 65 years, male gender, stage IB, lymphovascular invasion, perineural invasion, and elevated level of carcinoembryonic antigen were independent poor prognostic factors for recurrence-free survival of stage I gastric cancer. In Japan, research revealed that age≥70 years and lymphatic and/or venous invasion were independent prognostic factors for poor recurrence-free survival of T1N+ or T2-3N0 gastric cancer.^[9]^ Another study in Japan proved a location in the upper third of the stomach, tumor size of ≥30 mm, undifferentiated adenocarcinoma and clinical tumor depth were identified as independent risk factors for T1N0 gastric cancer.^[10]^ In China, from 734 early gastric carcinoma radical resections found that Independent risk factors for lymph node metastasis in early gastric carcinoma include tumor size ≥3.0 cm, submucosa invasion to a depth more than 200 μm, moderate/poor differentiation, lymphovascular invasion and tumor necrosis.^[11]^ Baesed on a joint study of China and the United States found that 7% (76) of our 1,058 patients from the United States (n = 414) and China (n = 644) recurred. Liver (43%) was the most common site of recurrence in both countries (US: 24%, China: 52%), followed by peritoneum (16%), lymph nodes (10%), and anastomosis (8%). Median time to recurrence was 23 months (US: 30 months, China: 23 months), which decreased with increasing T-stage (T1a: 27 months, T1b: 24 months, T2: 22 months). Tumor size (*P* = .001), depth of invasion (*P* = .010), histological type (*P* = .022) and lymphovascular invasion (*P* = .001) were independently associated with recurrence.^[12]^ As far as advanced gastric cancer, many researcher explored the related factors of recurrence of advanced gastric cancer. In Japan, a retrospective study^[13]^ showed that the 5-year relapse-free survival (RFS) rates of patients with Stage III gastric cancer were 42.0%. Univariate and multivariate analyses for RFS revealed that venous invasion was an independent factor predicting a shorter RFS. In China, another retrospective study^[14]^ displayed that tumors located at upper, middle third, or mixed, a positive lymph node ratio ≥0.335, pTNM stage III, lymphocyte count < 1.5 × 109/L, postoperative infection complications and adjuvant chemotherapy <6 cycles were all independent predictors for early recurrence after curative resection of stage II/III gastric cancer. The other 1 investigation in China^[15]^ about prognostic factors and recurrence patterns in T4 gastric cancer patients after curative resection indicated that age, location of tumor and intraoperative blood loss were independent prognostic factors for overall survival. After a median follow-up of 25.87 months, a total of 109 (43.8%) patients suffered from recurrence, and 90 patients had been observed specific recurrence sites, among which peritoneal metastasis was the most common recurrence pattern, 59.0% for T4a and 88.3% for T4b, respectively.

Another research hotspot concerning recurrence of gastric cancer concentrated upon early recurrence. In China, through a total of 149 patients with recurrence of gastric cancer/adenocarcinoma of the esophagogastric junction after curative resection proved that perineural invasion, postoperative chemotherapy and postoperative complications were independent factors associated with early recurrence after curative resection. The survival analysis showed that perineural invasion (*P* = .011) and postoperative complications (*P* = .007) were independent prognostic factors.^[15]^ Another study in China by 417 gastric cancer patients exhibited that there was no significant difference in recurrence patterns between early and late recurrence (*P* < .05 each). For pT1 stage gastric cancer, tumor size (*P* = .011) and pN stage (*P* = .048) were associated with early recurrence of gastric tumors. Patient age, pT stage, pN stage, Lauren histotype, lymphovascular invasion, intraoperative chemotherapy, and postoperative chemotherapy were independent predictors of early recurrence in patients with pT2-4a stage gastric cancer.^[16]^ A retrospective study in Japan^[17]^ via 96 patients with pStage III found that lymph node metastasis≥14, CA19-9≥37IU/mL and blood loss ≥445 mL were independent risk factors for early Recurrence after curative gastrectomy in pStage III GC. Another research in Japan showed that^[18]^ early recurrence was associated with a high lmph node ratio (*P* = .0020) and high CA19-9 levels (*P* = .0415). The other factors were not significantly associated with early recurrence. Lymph node ratio≥0.15 and CA19-9≥37 U/mL were effective surrogate markers for predicting early recurrence.

In short, little attention paid to middle and long term recurrence of gastric cancer. Moreover, the sample capacity of current study of risk factors for recurrence is small, in our present study, the 1-year survival rate was 94.14%, the 3-year survival rate was 69.53% and the 5-year survival rate was 37.71% respectively. We noticed that from 1 year to 3 years, the survival rate dropped nearly a quarter. What is more, from 3 year to 5 years, the survival rate dropped nearly a half. If we attached much importance to insure chemotherapy cycle adequately performance, will the overall survival rate may be significantly improved. At the same time, maximum diameter of tumor can be reduced through complete chemotherapy implement. Finally, overall survival ratio of follow-up within 3 years may be enhanced by this measure.

Futhermore, studies on the relationship between chemotherapy and recurrence of gastric cancer as following. A study in Japan focused on comparison of recurrence patterns between S-1 single drug and simple operation groups.^[19]^ The result was that overall and recurrence-free survival were better for the S-1 adjuvant group. In the surgery alone group, carcinoembryonic antigen ≥5 ng/mL, total gastrectomy, vessel invasion, pT4, and stage 3 were identified as significant prognostic factors. In striking contrast, macroscopic tumor size ≥50 mm was the only significant prognostic factor for the S-1 adjuvant group. Another study in Japan took notice of 34 patients underwent curative conversion surgery.^[20]^ The result was that in 17 (50%) patients, with a median time to recurrence of 22 months (range = 1–98 months). In 9 (53%) patients with recurrence, the pattern was consistent with their initial metastatic disease. Initial clinical T4b disease was the only significant independent risk factor affecting recurrence-free survival. The other 1 study in Japan through 396 patients proved the relationship between S-1 monotherapy and the timing and sites of recurrence.^[21]^ The results were that the 1-, 3- and 5-year RFS rates were 67.2%, 23.0% and 5.7%, respectively. Local recurrence, lymph node involvement and peritoneal and hematogenous metastases were found in 6, 25, 63, and 42 patients, respectively. Local recurrence and lymph node metastasis plateaued 3 years after gastrectomy. Peritoneal and hematogenous metastasis increased within 5 years after surgery. In patients with hematogenous metastasis, the number of liver metastases plateaued but increased in others. A study in Korea enrolled 130 patients who had undergone an R0 resection and had completed 6 cycles of adjuvant chemotherapy.^[22]^ The lymph node ratio (LNR), which was defined as the number of metastatic lymph nodes divided by the retrieved lymph nodes, was a significant risk factor in the lymph node-positive group (*P* < .01). Baseline CA19-9 level was a risk factor in the lymph node-negative group (*P* = .01).

We noticed that the sample studies on the relationship between chemotherapy and recurrence of gastric cancer was small. What is more, we need more samples and timing supervision to confirm the relationship between chemotherapy and recurrence of gastric cancer. At the same time, identify the effect of follow-up time on recurrence.

## 5 Conclusions

TNM Stage, chemotherapy cycle were closely related to recurrence of follow-up within 3 years. More than 9 cycles of chemotherapy was able to reduce the probability of recurrence. T Stage, TNM stage, maximum diameter of tumor were independent factors associated with overall survival of gastric cancer of follow-up within 3 years. About maximum diameter of tumor, the probability of death of more than 6 cm was 1.317 times less than 6 cm after surgery.

## Acknowledgments

Special thanks were given to Suwen Ji, Chenjie Fan for proofreading the patient follow-up information.

## Author contributions

**Writing – original draft:** Yifan Li.

**Writing – review & editing:** Yifan Li, Haoliang Zhao.
